# A multifactorial analysis of *in vitro* fertilization outcomes

**DOI:** 10.1530/RAF-25-0083

**Published:** 2026-03-26

**Authors:** Bahman Afsari, Snigdha Gollapudi, Aileen Portugal, Rand El Sharaiha, Megan Gornet, Dan I Lebovic, Patricia Jimenez, Kenan Omurtag, Ali Ahmady, Arash Darafsheh

**Affiliations:** ^1^ HIV and AIDS Malignancy Branch, Center for Cancer Research, National Cancer Institute, Bethesda, Maryland, USA; ^2^ Department of Radiation Oncology, WashU Medicine, St. Louis, Missouri, USA; ^3^ Division of Reproductive Endocrinology & Infertility, Department of Obstetrics & Gynecology, WashU Medicine, St. Louis, Missouri, USA

**Keywords:** age, blastocyst formation rate, IVF outcome, live birth, mature oocyte

## Abstract

**Abstract:**

Outcomes of *in vitro* fertilization (IVF) are woven by a complex interplay of multiple factors. Identifying how various variables correlate with the outcome of each step during the IVF procedure can inform on the overall success of the procedure. We studied 1,736 non-donor IVF cycles at our fertility center to evaluate associations between maternal age, body mass index (BMI), anti-Müllerian hormone (AMH) level, and antral follicle count (AFC) with the overall success of IVF in the first cycle, subsequent cycles, and all cycles, defined in terms of oocytes retrieved, oocyte maturation rate, fertilization rate, and live birth. The results indicated that maternal age was the strongest factor in IVF success with live birth rate of 57, 41, and 53% in the first cycle, subsequent cycles, and all cycles, respectively, in women < 35 years, and 27, 16, and 22% in the first cycle, subsequent cycles, and all cycles, respectively, in women 41–42 years old. AMH levels and AFC were strongly associated with oocyte retrieval and blastocyst formation but were less associated with the live birth. The number of mature oocytes and oocyte maturation rates were significantly associated with live birth. BMI did not show a significant correlation with oocyte yield and embryo development except in extremely obese patients. These findings support the use of individualized treatment strategies and development of predictive modeling based on biological markers to optimize IVF success.

**Lay summary:**

This study analyzed the data collected from the patients undergoing IVF to investigate the association between maternal age, anti-Müllerian hormone (AMH) – a protein hormone produced by the cells surrounding the developing eggs – levels, antral follicle count (AFC) – a measure of ovarian reserve or potential fertility, and BMI, and key reproductive outcomes including retrieved eggs, maturation rate, embryo development rate, and live birth. An analysis of multiple factors and IVF outcomes showed that maternal age remains the most significant factor of the IVF success. Although AMH and AFC are strong markers of ovarian response, their association with live birth is limited. Additionally, BMI did not show a significant impact on the outcomes.

## Introduction

Infertility, defined as failure to conceive after one year of regular unprotected intercourse, impacts approximately 17.5% of adults worldwide according to the World Health Organization (WHO) (World Health Organization 2023). Addressing infertility is an important component of sexual and reproductive health and rights (World Health Organization 2023); assisted reproductive technologies (ARTs) have emerged as a transformative solution to address this issue (Allahbadia *et al.* 2020, Jain & Singh 2023). *In vitro* fertilization (IVF) – for which Robert Edwards received the 2010 Nobel Prize in medicine for his contribution to the field – is responsible for 99% of ART procedures (Allahbadia *et al.* 2020, Jain & Singh 2023). Since the birth of Louise Brown, the first baby conceived through IVF in 1978 (García-Ferreyra *et al.* 2021), more than 12 million babies have been born using ARTs. Despite major advances in culture media (Cimadomo *et al.* 2018), vitrification (Hendriks *et al.* 2005, Broer *et al.* 2014), and genetic screening (Fleming *et al.* 2015, Practice Committee of the American Society for Reproductive Medicine 2020), success rates remain limited. Clinical pregnancy rates average around 40% for women under 35 and drop to ∼11% for women over 40, depending on factors such as maternal age, reproductive history, and lifestyle (Shrestha *et al.* 2015).

The main reason for the decline in successful pregnancies with age is the ovarian reserve, oocyte quality, and endometrial receptivity, which significantly affects the IVF success rate (Cimadomo *et al.* 2018). To assess the ovarian reserve and predict the response to ovarian stimulation, biomarkers such as anti-Müllerian hormone (AMH) and antral follicle count (AFC) are widely used (Hendriks *et al.* 2005, Broer *et al.* 2014). AMH, secreted by granulosa cells, acts as an indicator of the follicular pool that is remaining and is correlated with the oocyte retrieval rates during the IVF cycles (Practice Committee of the American Society for Reproductive Medicine 2020). Similarly, to get direct insights into the follicular recruitment and the ovarian response, AFC is used, which is determined via transvaginal ultrasound (Fleming *et al.* 2015). These biomarkers are essential in providing personalized IVF protocols based on the individual and help in clinical decision-making. Additionally, lifestyle and metabolic factors, such as body mass index (BMI), may influence IVF success. Obesity has been associated with lower birth rates, poorer oocyte quality, and increased miscarriage risk emphasizing the need to understand the effects on IVF outcomes (García-Ferreyra *et al.* 2021). Although these factors have been examined individually, their combined impact on clinical IVF outcomes in routine practice settings remains insufficiently characterized.

Identifying and understanding how various variables influence the outcome of each step during the IVF procedure can inform the overall success of the procedure. This retrospective study analyzes clinical dataset from 1,736 IVF cycles conducted at a single fertility clinic, WashU Medicine, over ten years. By examining the combined impact of age, AMH, AFC, and BMI on key outcomes – including oocyte retrieval, maturation rate, blastocyst formation, and live birth (LB) – our study serves as a valuable counseling tool, offering a more comprehensive and integrated assessment of IVF outcomes.

## Materials and methods

### Regulatory and ethical approval

This retrospective study was approval by the Institutional Review Board of WashU Medicine (IRB 202202106). Patients’ information was de-identified prior to analysis.

### Study design

This study included patients who underwent controlled ovarian stimulation, oocyte retrieval, and subsequent ART treatment using IVF between January 2014 and December 2023 at a single fertility center affiliated with WashU Medicine. Data include treatment cycle number, age, BMI, AFC, AMH level, and the related outcomes defined in the next section. Out of 3,871 treated patients, 1,859 patients had full information; among them patients with ovarian disorder, diminished ovarian reserve, severe male factor, preimplantation genetic testing (PGT) results, and donor oocytes were excluded from data analysis. In addition, the 16 remaining patients >42 years were excluded due to insufficient sample size for statistical analysis. As a result, 1,411 patients with 20–42 years old were included in the data analysis. A total of 1,736 IVF/ICSI cycles were analyzed. All patients were treated with medical protocols based on their infertility diagnosis. The medical protocols included the long GnRH agonist protocol, antagonist, Lupron flare, letrozole, luteal E2 antagonist, luteal E2 flare, and others (Shrestha *et al.* 2015). We have sub-divided the data into three groups: IVF cycle #1, subsequent cycles, and all cycles. We performed analysis separately on the cycle #1’s data, subsequent cycles’ data, and all cycles’ data for comparative analysis of first-time versus repeated treatment cycles offering insights into factors affecting the pregnancy outcomes.

The primary factors analyzed included patient age, BMI, AFC, and serum AMH level (most recent value relative to cycle started). Outcomes of interest included the number of oocytes retrieved (OR), number of mature oocytes (MOs), number of normally fertilized oocytes (2PNs), number of blastocysts, and LB. In addition to absolute counts, we assessed the maturation rate, defined as the ratio of the number of MOs by the number of OR and blastocyst formation rate (blastocysts/2PNs) to evaluate oocyte quality and developmental progression. The outcome was LB after the clinical pregnancy was confirmed.

The BMI values for each patient were collected. To have a better understanding of how it might correlate with the IVF outcomes, these values were categorized into six groups following the WHO guidelines (World Health Organization 2000). Patients were classified as underweight (BMI < 18.5), normal weight (18.5–24.9), overweight (25.0–29.9), class 1 obesity (30.0–34.9), class 2 obesity (35.0–39.9), and extreme obesity (≥40).

### IVF workflow description

**Figure 1:**
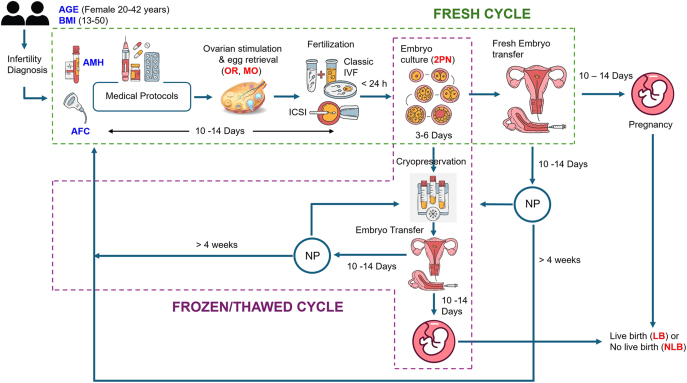
Schematic illustration of the IVF process workflow, starting with infertility diagnosis, followed by ovarian stimulation, egg retrieval, fertilization, and embryo culture. The chart highlights both fresh cycles, where embryos are transferred directly, and frozen/thawed cycles, where cryopreserved embryos are later thawed and transferred. Outcomes indicated by red font include LB, OR, MOs, and normally fertilized oocyte (2PN) counts tracked throughout the process. Key metrics used in the analysis, indicated by blue font, are the age, BMI, anti-Müllerian hormone (AMH) levels, and antral follicle count (AFC). 2PN, two pronuclei (fertilized oocytes); NP, no pregnancy (cycle restart).

Figure 1 illustrates the workflow of the IVF procedure. Before starting ovarian stimulation, the AMH levels were measured through blood work, and the AFC was assessed via transvaginal ultrasound to evaluate ovarian reserve. Ovarian stimulation lasts 7–14 days, followed by a trigger injection of hCG or GnRH agonist to induce final oocyte maturation. Moreover, advancements in protocols such as the ‘dual trigger’ approach, which combines hCG with GnRH agonist, have shown promising results in enhancing oocyte yield and maturation rates, particularly in patients at risk for ovarian hyperstimulation syndrome (OHSS) (de Oliveira *et al.* 2016). Oocyte retrieval was performed approximately 34–36 h post-trigger, and MOs were identified under an inverted light microscope. The method of retrieval typically involved transvaginal ultrasound guidance, allowing for the collection of multiple oocytes from the follicles that have developed during stimulation (Lawrenz *et al.* 2024). Fertilization occurred via conventional IVF or intracytoplasmic sperm injection (ICSI), with embryos monitored for 3–6 days. ICSI was performed when the first cycle was not successful (low fertilization). In a fresh cycle, one or more embryos were transferred on day 3 or day 5, while viable embryos were cryopreserved through vitrification for future use called the frozen cycle. The blastocyst formation rate is the proportion of blastocysts from normal fertilized oocytes (2PN). Pregnancy was confirmed through a serum beta-hCG test 10–14 days post-transfer. If unsuccessful, a frozen embryo transfer (FET) cycle can be initiated after at least one full menstrual cycle.

### Definition of outcome

Four parameters were considered as outcome: number of OR was defined as the total number of oocytes obtained during oocyte retrieval, while MOs referred to the number of metaphase II (MII) oocytes identified within two days post-retrieval. Normally fertilized oocytes (2PNs) were defined as zygotes displaying two pronuclei approximately 16–18 h post-insemination, confirming successful fertilization. Embryo development was assessed by evaluating the number of blastocysts, defined as the total number of embryos that reached the blastocyst stage between days 5 and 7 after fertilization. The blastocyst formation rate was calculated as the number of blastocysts divided by the number of normally fertilized oocytes, LB defined as a baby that was born after 24 full weeks of gestation and had positive vital signs. No live birth (NLB) indicates cases where the patient experienced miscarriage, stillbirth, ectopic pregnancy, elective abortion, or spontaneous pregnancy reduction.

### Statistical analysis

All statistical analyses were conducted using R (version 4.5.1). Data processing and visualization were performed using dplyr (1.1.4), stats (4.5.1), and tidyr (1.3.2), ggplot2 (4.0.1), and ggpubr (0.6.2).

Descriptive statistics were used to summarize outcomes by the age group and cycle number. To assess differences in cardinal variables (e.g. number of OR, MOs, and 2PNs) across age groups, we applied the independent two-sample *t*-tests or Wilcoxon rank-sum tests, depending on the distribution of the data. For categorical comparisons such as LB rates, Fisher’s exact test was used. Additionally, linear regression analysis was performed to assess the relationship between age and reproductive outcomes (number of OR, MOs, and 2PNs). Regression coefficients, *P*-values, and *R*^2^ values were reported to describe the direction and strength of associations. We analyzed the data in samples only in cycle #1, subsequent cycles, and samples in all cycles. By studying sample cycle #1 separately, we intended to remove ‘survivorship bias’, which might be introduced due to the failure of the previous cycles (Szklo & Nieto 2004, Woodward 2004, Rothman *et al.* 2008). Once we established a pattern in cycle #1, we test if the pattern holds for the following cycles.

## Results

**Figure 2:**
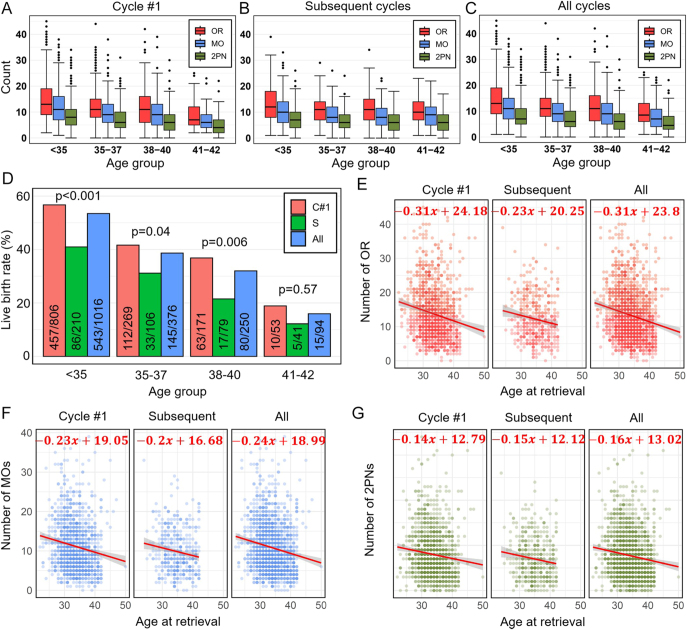
Number of OR, MOs, and normally fertilized oocytes (2PNs) in (A) cycle #1, (B) subsequent cycles, and (C) all cycles as a function of the age group. (D) Percentage of LBs by age group for cycle #1 (C#1), subsequent cycles (S), and all cycles (all). Numbers in each bar represent the number of LBs and numbers of women in each group. Scatter plot of number of (E) OR, (F) MOs, and (G) 2PNs as a function of age at retrieval along with the corresponding regression lines.

Figure 2A, B, C shows the number of OR, MOs, and 2PNs as a function of the age group for cycle #1, subsequent cycles, and all cycles, respectively. Figure 2C shows the LB percentage as a function of the age group corresponding to cycle #1, subsequent cycles, and all cycles. The analysis of reproductive outcomes across cycle #1, subsequent cycles, and all cycles reveals a clear trend of declining success with increasing age. Patients under 35 demonstrated the highest median values for the key metrics defined as the outcome in this work. In cycle #1, patients under 35 had the highest outcomes, with a median of 13 OR (IQR: 9–19), 11 MOs (IQR: 7–16), and 8 2PNs (IQR: 5–11). In contrast, patients in the 41–42 years age group had a median of 7 OR (IQR: 5–12), 6 MOs (IQR: 4–9), and 4 2PNs (IQR: 2–7). In subsequent cycles, patients < 35 had a median of 12 OR (IQR: 8–18), 10 MOs (IQR: 6–14), and 7 2PNs (IQR: 4–10). Among those in 41–42 years old group, the corresponding medians declined to 10 OR (IQR: 7–14), 9 MOs (IQR: 5–12), and 6 2PNs (IQR: 3–9). In all cycles, patients < 35 had a median of 13 OR (IQR: 9–19), 11 MOs (IQR: 7–15), and 7 2PNs (IQR: 5–11). Among those in 41–42 years old group, the medians declined to 8.5 OR (IQR: 6–13), 7 MOs (IQR: 4–10.75), and 4.5 2PNs (IQR: 3–8). These differences were statistically significant in all cycle #1 and all cycles (Wilcoxon *P* < 0.001 for OR, MO, and 2PN), while in the subsequent cycles, the differences were not statistically significant due to the insufficient sample size (Wilcoxon *P* > 0.1 for OR, MO, and 2PN). These findings emphasize the impact of ovarian aging on fertility, as both the quantity and quality of oocytes diminish with age. The LB percentage by age group further underscores this decline. LB rates for the first cycle, subsequent cycles, and all cycles were <35 years (57, 41, and 53%), 35–37 years (42, 31, and 39%), 38–40 years (37, 22, and 32%), and 41–42 years (19, 12, and 16%), respectively. The differences across age groups were statistically significant, as confirmed by Fisher’s exact test (*P* < 0.001).

Further analysis of age and OR through scatter plot regression analysis presented in Fig. 2E reveals a loose negative correlation, showing that for each additional year of age, OR decreases by approximately 0.38 oocytes (*P* < 0.001, Pearson correlation = −0.2) in cycle #1. As can be seen, there is a relative noticeable variation at any given age. A similar decline is observed in the number of MOs (Fig. 2F), with a reduction of 0.3 oocytes per year (*P* < 0.001, Pearson correlation = −0.18). Figure 2G shows that the number of 2PNs formed declines by 0.2 per year (*P* < 0.001, Pearson correlation = −0.14). A similar behavior is seen for subsequent and all cycles.

**Figure 3:**
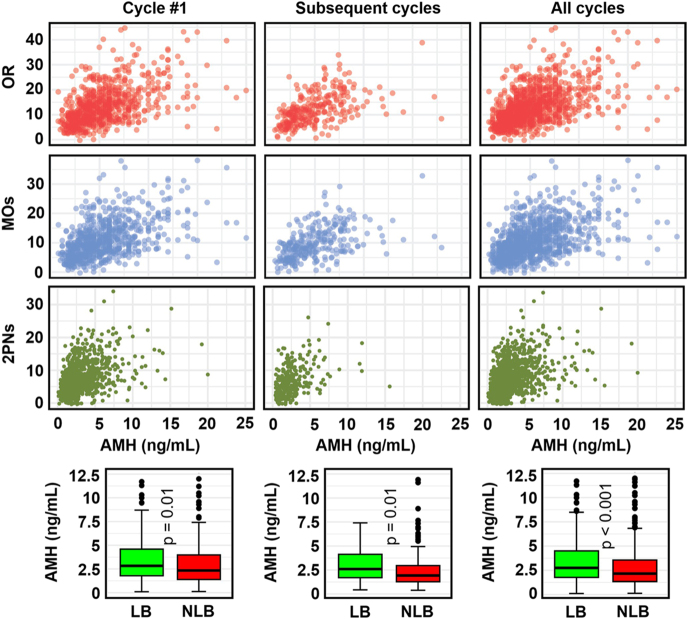
Relationship between the AMH level (last value) and the number of OR (top row), MOs (second row), and 2PNs (third row). The bottom panels compare the AMH level (last value) for patients with LB or NLB. Cycle #1: left column; subsequent cycles: middle column; all cycles: right column.

AMH, a key marker of ovarian reserve, was positively associated with higher numbers of OR, MOs, and 2PNs, particularly in the range of 0–10 ng/mL (Fig. 3). Median AMH levels were higher in LB compared with NLB groups in cycle #1, subsequent cycles, and all cycles (Wilcoxon *P*-values <0.001); however, these differences are not dramatic: In cycle #1, the median AMH was 2.82 ng/mL (IQR: 1.79–4.58 ng/mL) in LB vs 2.38 ng/mL (IQR: 1.4–4 ng/mL) in the NLB group; in subsequent cycles, it was 2.62 ng/mL (IQR: 1.72–4.16 ng/mL) in LB vs 1.9 ng/mL (IQR: 1.27–2.95 ng/mL) in the NLB group; and in all cycles, it was 2.75 ng/mL (IQR: 1.77–4.5 ng/mL) in LB vs 2.16 ng/mL (IQR: 1.34–3.58 ng/mL) in the NLB group.

**Figure 4:**
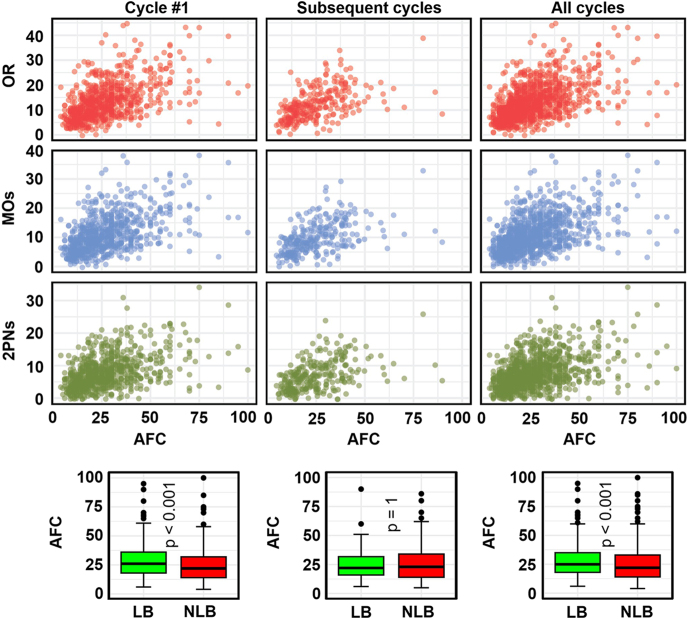
Relationship between AFC and the number of OR (top row), MOs (second row), and 2PNs (third row). The bottom panels compare AFC for patients with LB or NLB. Cycle #1: left column; subsequent cycles: middle column; all cycles: right column.

AFC, another key ovarian reserve marker, was positively associated with oocyte retrieval, maturation, and fertilization outcomes (Fig. 4). The strongest increases were observed for AFC values below 30. These patterns remained consistent in cycle #1, subsequent cycles, and all cycles, though with slightly reduced variability. Unfortunately, AFC was not measured for 405 samples, which reduced our statistical power for further analysis, especially for subsequent cycles, which only had 279 samples. Patients who achieved LB had statistically significantly higher AFC values than those who did not in cycle #1 and all cycles for which we had enough samples. Overall, the median AFC in the LB group was 25 (IQR: 18–35) compared to 22 (IQR: 14–33) in the non-LB group. This trend is not persistent across age groups: for example, among patients <35 years, the median AFC for those with LB was 28 (IQR: 19–38) in cycle #1 and in all cycles, compared to 26 (IQR: 16–37.5) in cycle #1 and all cycles in those without LB. In the other age groups, the differences between LB and non-LB were not statistically significant, though.

**Figure 5:**
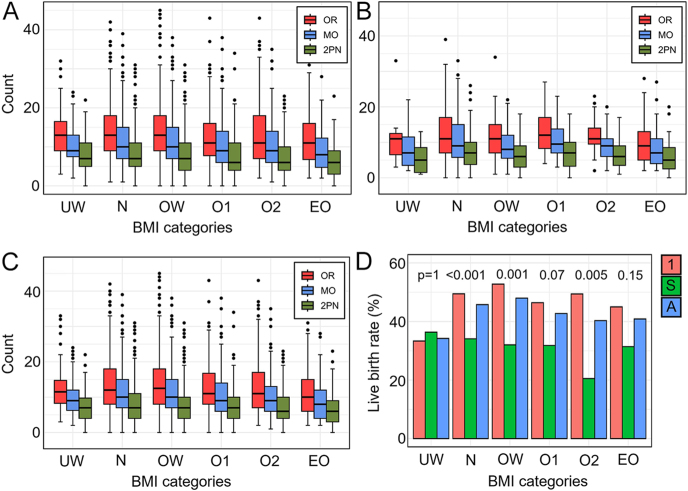
(A, B, C) Distribution of OR, MOs, and 2PNs (fertilized zygotes) across BMI categories for cycle #1, subsequent cycles, and all cycles, respectively. (D) LB rate across BMI categories for cycle #1 (1), subsequent cycles (S), and all cycles (A). UW, underweight (BMI < 18.5); N, normal weight (18.5–24.9); OW, overweight (25.0–29.9); O1, class 1 obesity (30.0–34.9); O2, class 2 obesity (35.0–39.9); EO, extreme obesity (≥40).

Analysis of BMI-based influences did not reveal any statistically significant differences between BMI groups and their subsequent BMI group in LB status using Fisher’s exact test, as shown in Fig. 5. Across cycle #1, subsequent cycles, and all cycles, the median values for OR and 2PNs remained largely consistent across BMI categories. In cycle #1, patients in the normal BMI group had a median of 13 OR (IQR: 9–18), compared with 11 in the extreme obesity group (IQR: 7–16). Across all cycles, the medians were 12 (IQR: 8–18) and 10 (IQR: 6–15), respectively. The median MO was 10 (IQR: 7–15) for normal BMIs, compared to 8 (IQR: 4–12) for the extreme obesity group. The median 2PN in the normal BMI was 7 (IQR: 5–11), slightly higher than that in the extremely obese group (6 (IQR: 4–10)). These differences between normal and extreme obese categories are statistically significant (Wilcoxon *P*-value <0.001), while in terms of effect size, the differences are negligible (Cohen’s d < 0.33). LB rates also showed no significant differences across BMI groups (*P* = 0.56). These findings suggest that BMI showed some statistical association with ovarian response and fertilization, and the effect size was consistently small, limiting clinical relevance.

**Figure 6:**
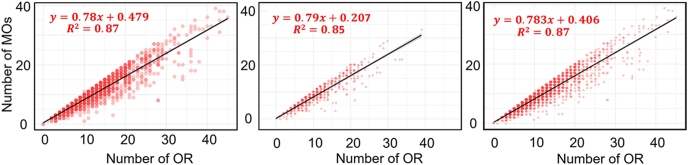
The relationship between the number of OR and the number of MOs, with a linear regression line for cycle #1 (left), subsequent cycles (middle), and all cycles (right).

Figure 6 shows the number of MOs as a function of OR illustrating the efficiency of the maturation process. Across cycle #1, subsequent cycles, and all cycles, there was a strong positive correlation, indicating that approximately 80% of retrieved oocytes matured successfully. This consistency underscores the robustness of the oocyte maturation process, irrespective of the age or cycle type.

**Figure 7:**
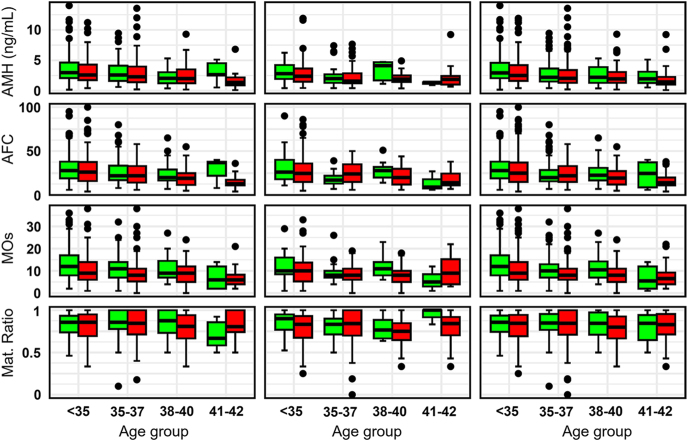
AMH (last value) (top row), AFC (second row), number of MOs (third row), and maturation ratio (bottom row) by age group stratified by LB category (LB: green; no LB: red) for cycle #1 (left column), subsequent cycles (middle column), and all cycles (right column).

When exploring the interplay between ovarian reserve markers and LB outcomes, a clear association was observed as shown in Fig. 7. Across all IVF cycles, patients who achieved LB had higher AFCs (median: 25 vs 22, mean: 28.4 vs 25.3, Wilcoxon and *t*-test *P* < 0.001) and AMH levels (median: 2.7 vs 2.15 ng/mL, mean: 3.5 vs 2.77 ng/mL, Wilcoxon and *t*-test *P* < 0.001) compared to those without a LB. These findings indicate the association of AFC and AMH with LB outcomes across IVF cycles with the strongest association observed in younger patients.

The analysis of MOs as a function of LB outcomes revealed similar trends. Among patients under 35, those achieving LBs had a median of 12 MOs in cycle #1 and 12 across all cycles, compared with 9 and 9 for unsuccessful outcomes, respectively. This trend persisted across age groups, with medians declining with age. In the 41–42 age group, LB outcomes were associated with a median of 7 MOs in cycle #1 and 8 MOs across all cycles, compared to 6 and 7 MOs for unsuccessful outcomes, respectively. The differences in lower ages are statistically significant (*P* < 0.001), but for higher ages, the differences are not significant due to low sample size. Maturation rates did not display a correlation with LB outcomes. Although maturation rates declined slightly with age, patients who achieved LBs had slightly higher, but statistically insignificant, median maturation rates across all age groups, with younger groups reaching rates close to 0.84.

## Discussion

Our findings provide valuable insights into age-related declines in reproductive outcomes. We observed a decrease in OR, MOs, and normally fertilized oocytes (2PN ICSI + Insem) with increasing age. Patients under 35 years old had a median of 13, 12, and 13 OR across cycle #1, subsequent cycles, and all cycles, respectively, which declined to 7, 10, and 8.5 in those between 41 and 42 years old. This trend is consistent with findings from Romanski *et al.* (2022), where a gradual decline in oocyte retrieval from a median of 18 in patients ≤30 years old to 10 in those between 41 and 42 years old was reported, reinforcing the association between the age and diminished ovarian response. The study of Kidera *et al.* corroborates this trend, noting higher fertilization rates in younger maternal age groups and a significant decline in clinical pregnancy and LB rates in patients > 40 years (Kidera *et al.* 2022). Havrljenko *et al.* further emphasized the decline, with LB rates dropping from 38.2% in younger groups (≤35) to 8% in the oldest (≥45) groups, while miscarriage rates increased with age (Havrljenko *et al.* 2023). These findings align with our observation that both the quantity and quality of oocytes are significantly affected by ovarian aging.

The association between AMH and AFC with ovarian response was reaffirmed in our study. We observed that the number of OR increased from approximately median 11 (IQR: 7–15) oocytes in patients with AMH < 5 ng/mL to median 19.5 (IQR: 13–28.5) oocytes in those with AMH > 10 ng/mL. We also observed that patients younger than 35 had the highest average AFC (29.6), which decreases progressively with age, dropping to 17.3 in patients in 41–42 range. Kozlowski *et al.* reported a positive correlation between AMH and oocyte yield, with retrieved oocytes increasing from 5.42 at AMH 1.35 ng/mL to 9.5 at AMH 2.65 ng/mL, further supporting AMH as a strong indicator of ovarian reserve (Kozlowski *et al.* 2022). Poulain *et al.* corroborate this with a positive correlation between AMH and AFC, observing mean ratios of 6.6 ± 4.7 (Oc/AFC) and 0.7 ± 0.4 (Oc/AMH) (Poulain *et al.* 2021). However, Fanton *et al.* emphasize diminishing returns in cumulative LB rates once oocyte retrieval exceeds 20, aligning with our findings that while AMH and AFC contribute to estimating ovarian response, their incremental benefits are limited beyond certain thresholds (Fanton *et al.* 2023).

The impact of blastocyst development rates and age was evident in our dataset, where blastulation rates declined from 50% in patients < 35 to 33.3% in patients with 41–42 years old (Wilcoxon *P*-value <0.02). This aligns with the result by Romanski *et al.* who observed stable rates through age 40, followed by a significant decline at ≥41 years (e.g., 42 years: 56.8–47.1%) (Romanski *et al.* 2022). Similarly, Sainte-Rose *et al.* reported the highest blastocyst formation rate (59%) in women aged 23–36, with a significant drop to 48% in women aged 41–43 (Sainte-Rose *et al.* 2021). These trends reinforce the age-related decline in blastocyst development. Additionally, Catherino *et al.* noted an increasing trend in ART utilization, particularly embryo and oocyte preservation, suggesting adaptation strategies to mitigate age-related fertility decline (Wirka *et al.* 2021). As Tur-Kaspa *et al.* previously demonstrated, oocyte maturity is a strong predictor of blastocyst development and euploidy rates across all age groups, with the exception of women > 42, where limited sample size reduces statistical significance (Tur-Kaspa *et al.* 2024). This aligns with our conclusion that while blastocyst formation diminishes with age, maturation efficiency remains relatively stable (∼0.8). These findings align with those of Houri *et al.* who reported a median maturation rate of approximately 80% in patients aged ≤ 38 years undergoing their first IVF cycle (Houri *et al.* 2023). This stability highlights the ability to maintain the oocyte maturation process despite the reduced quality of the aging oocyte.

Regarding BMI and IVF outcomes, our study found that BMI had no statistically significant impact on ovarian response across all BMI categories except in extremely obese patients in which slightly decreased outcomes were noted compared to the normal BMI group. Kluge reported a significant decrease in cumulative LB rate with increasing BMI, from 32.6% in normal-weight women to 7.6% in obesity class III, alongside increased risks of hypertensive disorders and preterm birth (Kluge *et al.* 2023). Sarais *et al.* found a significantly reduced percentage of MOs in obese patients (BMI: ≥ 30) compared to normal-weight women (BMI: 18.5–24.99), though ongoing pregnancy rates did not differ (Sarais *et al.* 2016). Correia *et al.* highlight that oocyte retrieval recommendations increase with BMI and age, recommending fewer oocytes for younger patients and more for older individuals (e.g. 7–9 oocytes for patients aged 32–37) (Correia *et al.* 2023). Ruiz *et al.* further demonstrated that natural cycles in the frozen embryo transfer (FET) yield better LB rates where women with class I/II obesity have pronounced negative effects [30]. These findings support our conclusion that BMI plays a secondary role compared to age and ovarian reserve indicators.

A unique aspect of our study was the comparison of cycle #1’s, subsequent cycles’, and all cycles’ outcomes. In our study, patients who went through their first cycle had a higher chance of success compared to those who had an unsuccessful cycle #1 and went through the next cycle. Furthermore, our study found that cumulative IVF cycles slightly improved retrieval rates, but age remained the dominant factor influencing reproductive success, with a cumulative LB rate (CLBR) of 49–54% across all cycles. Khalife *et al.* reported a CLBR of 33.0% for the first cycle, increasing to 56.9% after three cycles and 67.9% after six cycles, emphasizing the incremental benefit of repeated cycles, particularly in women younger than 35 (Khalife *et al.* 2020). Wang *et al.* further noted lower CLBRs (34.4%) in older patients compared to younger ones (68.3%), aligning with our findings that while cumulative cycles modestly enhance success, age remains the critical determinant of final reproductive success (Wang *et al.* 2023). The reason for higher LB rate in cycle #1 in our study compared to the other studies can be associated with the exclusion criteria used in our study. On the other hand, in our study, patients opted to proceed with additional cycles and had 2 cycles on average, which explains a smaller improvement in CLBR in our study compared to other studies in which patients underwent up to 6 cycles.

Centers for Disease Control and Prevention (CDC) reported that out of 246,087 ART cycles initiated in the US with the intent to transfer at least one embryo, only 91,906 live birth deliveries have occurred corresponding to 37% overall success rate (Centers for Disease Control and Prevention 2023). Similarly, a success rate of ∼30% has been reported by the European IVF-monitoring Consortium (De Geyter *et al.* 2020). ART data from Africa show a higher rate of 41.9% in autologous cycles, although regional disparities in access to advanced technologies may contribute to this variation (Archary *et al.* 2024). We observed a live birth rate of 54% over the whole population and cycles. Our LB rate is higher than those studies due to our exclusion criteria. Notably, our study did not consider donor cycles, allowing us to concentrate on the effects of the patient-specific factors. A study involving donor egg cycles has reported that there was a stable LB rate of about 40% across the age groups and slightly lower in women aged 50 years and older (54.2%) compared with those aged 45–46 years (58.0%), although these differences were not statistically significant (Chen *et al.* 2024).

Our study found that successful LB outcomes are linked to higher median AMH and AFC values, although the variability in these markers limits their predictive accuracy. Additionally, we observed a strong positive correlation (*r* = 0.93) between OR and MOs, reflecting the efficiency of the maturation process. Similarly, the findings of Fanton *et al.* highlight a linear relationship between OR, fertilized oocytes, and blastocysts, with cumulative LB rates plateauing beyond 16–20 retrieved oocytes (Fanton *et al.* 2023). Building on this, Jamil *et al.* reported that while oocyte retrieval positively correlates with blastocyst formation rate, excessive retrieval is associated with reduced maturation and fertilization rates (Jamil *et al.* 2022). They concluded that retrieving 6–15 oocytes offer the best outcomes, balancing embryo quality and minimizing risks such as ovarian hyperstimulation. Collection of data analyses can be useful in developing AI-based models to predict the outcomes (Fouks *et al.* 2025, Garg *et al.* 2025, Hall *et al.* 2025).

Our study provides a large-scale analysis of 1,736 IVF cycles performed over a 10-year period, offering comprehensive insights into key reproductive outcomes. By evaluating age, AMH, AFC, and BMI in first, subsequent, and cumulative cycles, we provide clinically relevant data to guide patient counseling and individualized treatment planning. Additionally, our study benefits from a single-center design, reducing variability in laboratory protocols and clinical decision-making. Despite these strengths, several limitations should be considered. Our study is retrospective, which introduces potential bias in patient selection and data completeness. Additionally, while we evaluated biomarkers of ovarian reserve (AMH, AFC) and oocyte yield, we did not assess embryo ploidy status, a key determinant of LB rates, in patients > 37 years of age. Our analysis also did not include donor oocyte cycles, limiting the generalizability of findings to patients using autologous oocytes. It also did not specifically investigate the role of number transferred embryos as well as fresh vs frozen cycles for the pregnancy and LB rate. While we explored BMI as a factor in IVF success, we did not examine other metabolic markers (e.g., insulin resistance and lipid profiles) that could further impact reproductive outcomes. Another limitation of this study is that our fertility center has a required BMI < 50 for undergoing ART. As a result, the findings may not be fully generalizable to populations with higher BMI values, as outcomes in these patients were not captured in this dataset.

## Conclusion

This study highlights the significant impact of maternal age on IVF outcomes, demonstrating that while AMH and AFC are strong indicators of ovarian response, their correlation with LB is limited. The number of blastocysts and LB rates decline with increasing age, indicating the critical role of oocyte quality alongside quantity. Additionally, BMI had minimal influence on oocyte yield and reproductive outcomes. These findings underscore the importance of individualized treatment strategies and developing predictive modeling to optimize ART success rates.

## Declaration of interest

The authors declare that there is no conflict of interest that could be perceived as prejudicing the impartiality of the work reported.

## Funding

BA was supported by the Intramural Research Program of the NIH.

## Author contribution statement

AD and AA contributed to the conception and design of the study. BA, SG, and AD analyzed the initial data. BA and AD analyzed the data presented in the revised manuscript. The manuscript was drafted by AD and SG. BA, SG, AP, RES, MG, DIL, PJ, KO, AH, and AD discussed the results and reviewed the manuscript.

## Data availability

Data are available from the corresponding author upon reasonable request.
